# Soluble HLA in the Aqueous Humour of Uveal Melanoma Is Associated with Unfavourable Tumour Characteristics

**DOI:** 10.3390/cancers11081202

**Published:** 2019-08-18

**Authors:** Annemijn P. A. Wierenga, Gülçin Gezgin, Els van Beelen, Michael Eikmans, Marijke Spruyt-Gerritse, Niels J. Brouwer, Mieke Versluis, Robert M. Verdijk, Sjoerd G. van Duinen, Marina Marinkovic, Gregorius P. M. Luyten, Martine J. Jager

**Affiliations:** 1 Department of Ophthalmology, Leiden University Medical Center, Albinusdreef 2, 2333 ZA Leiden, The Netherlands; 2 Department of Immunohematology and Blood Transfusion, Leiden University Medical Center, 2333ZA Leiden, The Netherlands; 3 Department of Pathology, Leiden University Medical Center, 2333ZA Leiden, The Netherlands; 4 Department of Pathology, Section Ophthalmic Pathology, Erasmus MC University Medical Center, 3015GD Rotterdam, The Netherlands

**Keywords:** uveal melanoma, soluble HLA, infiltrate, aqueous humour, liquid biopsies, immunotherapy

## Abstract

A high HLA expression in uveal melanoma (UM) is part of the prognostically unfavorable inflammatory phenotype. We wondered whether the presence of soluble HLA (sHLA) in the aqueous humour is associated with clinical, histopathological or genetic tumour characteristics, and represents tumour HLA expression and intratumoural inflammation. Aqueous humour from 108 UM patients was analysed for the presence of sHLA, using a Luminex assay specific for HLA Class I. Clinical and genetic parameters were compared between sHLA-positive and negative eyes. A qPCR analysis was performed on tumour tissue using a Fluidigm assay. In 19/108 UM-containing eyes, the sHLA level in the aqueous was above the detection limit. Tumours in sHLA-positive eyes were significantly larger, more frequently involved the ciliary body, and more often showed monosomy 3, gain of chromosome 8q and loss of BAP1 staining. Melanoma-related survival was worse in patients with sHLA-positive aqueous humour. sHLA in the aqueous did not represent the tumour’s HLA expression and did not relate to immune cell infiltration in the tumour. We conclude that UM-containing eyes may contain sHLA in the aqueous humour, where it is a prognostically-unfavourable sign and may influence local immune responses.

## 1. Introduction

Although rare, with an incidence varying between 5.0 per million in the United States of America to 9.5 per million in Northern Europe, uveal melanoma (UM) is the most common primary ocular malignancy in adults [1,2]. The metastatic route of UM is mainly hematogenous, with about 50% of UM patients developing metastases, which occur predominantly in the liver [3,4]. Important genetic prognostic parameters are the monosomy of chromosome 3 (M3), a gain of the long arm of chromosome 8 (8q) [5] and a mutation in the BRCA1-Associated Protein 1 (BAP1) gene [6,7,8,9,10].

Prognostically-infaust tumours often carry an inflammatory phenotype, which is characterised by infiltrating lymphocytes and macrophages and a high expression of HLA Class I [11,12,13]. The relation between a high HLA Class I expression in UM and an increased risk of developing metastases has been reported [11,12,14,15,16,17,18]. HLA expression is an important prerequisite for T cell-mediated immunotherapy, and information on a tumour’s HLA expression may therefore help to identify cases that are good candidates for T cell-mediated therapy; for example, with IMCgp100 (Immunocore gp100 TCR-based bispecific T cell redirector) [19]. 

Information regarding the HLA expression level of the tumour might be obtained by studying the non-cell bound or soluble version of HLA (sHLA) in the aqueous humour. The presence of soluble HLA was first found in plasma of healthy HLA-A2 positive individuals in a study in 1970 in Leiden, The Netherlands [20]. The sHLA complex consists of an α chain which is non-covalently bound to β2-microglobulin. Soluble HLA may result from shedding from the cell membrane, by proteolytic cleavage of membrane-bound proteins or from alternative splice variants, leading to a secreted version of sHLA molecules [21].

Since its discovery, sHLA has been determined in different body fluids, including blood, plasma, amniotic fluid, seminal plasma, synovial fluid, sweat, urine and peritoneal dialysate. Levels of sHLA have been studied in the context of acute and chronic graft-versus-host disease, preeclampsia, rheumatoid arthritis and chronic renal failure [22,23,24,25,26]. In patients with varicella meningitis, sHLA was detected in the cerebrospinal fluid (CSF); in patients with AIDS, the concentration of sHLA in both serum and CSF directly correlated to the stage of the disease, with a higher concentration in more advanced cases [27]. Levels of circulating sHLA Class I molecules differ per person based on the presence of disease, as well as the inherited HLA haplotype [28]. sHLA may have immunomodulatory effects [29]: The level of sHLA correlates to the progression rate of several malignant diseases, and the shedding of sHLA molecules is considered a mechanism for evasion by tumour cells from immune recognition [30].

For enucleated UM patients, prognostic information comes from the tumour’s size, and its chromosome and mutation status. Knowledge regarding the immune status of the tumour and its HLA expression may one day be useful in deciding whether immunotherapy may be appropriate as adjuvant therapy to prevent metastases. 

We developed and optimised an assay to assess sHLA Class I in small amounts of anterior chamber fluid and determined whether we can detect this in the aqueous humour of UM-containing eyes. The assay recognises the soluble version of HLA Class I. Our data show that it is possible to determine sHLA Class I levels in the aqueous humour of UM eyes, and that the presence of sHLA is related to survival, but that this is more related to the presence of a large, anteriorly-located tumour than to the expression of HLA on the tumour cells.

## 2. Results

### 2.1. Patient Demographics

A high expression of HLA antigens on UM cells is an indicator of inflammation and of a bad prognosis. We wondered whether analysis of sHLA molecules in aqueous humour can be used to indirectly determine HLA expression as an indicator of the presence of an inflammatory phenotype in the tumour, and whether sHLA expression in the aqueous humour is higher in eyes with a high-risk UM. Using a newly-developed Luminex assay, we measured sHLA in aqueous humour samples of 108 UM-containing eyes and observed measurable levels in 19/108 (18%). We compared the characteristics of these 19 sHLA-positive cases with those of the 89 sHLA-negative cases (see Table 1 for patient and tumour characteristics).

The tumours in the sHLA-positive group were thicker, with a mean tumour prominence of 9.8 mm (SD = 2.2 mm) versus a mean prominence of 7.0 mm (SD = 2.9 mm) in the sHLA-negative group (*p* < 0.001), and more often involved the ciliary body (*p* < 0.001). Tumours from the sHLA-positive group belonged to higher American Joint Committee on Cancer (AJCC) stages (*p* < 0.001).

With regard to chromosome status, the sHLA-positive group showed significantly more often monosomy 3 (15/18, 83%) compared to the sHLA-negative group (48/85, 57%) (*p* = 0.034). Additionally, the sHLA-positive group more frequently showed gain of 8q (16/17, 94%) as opposed to the sHLA-negative group (43/78, 55%, *p* = 0.003), but no difference was observed with regard to chromosome 6p (*p* = 0.98). A total of 16/18 (89%) of the tumours in the sHLA-positive group had lost BAP1 staining, which was significantly different from the 50/79 (63%) tumours in the sHLA-negative group (*p* = 0.036).

### 2.2. sHLA in Aqueous Humour Is Related to Survival

**Figure 1:**
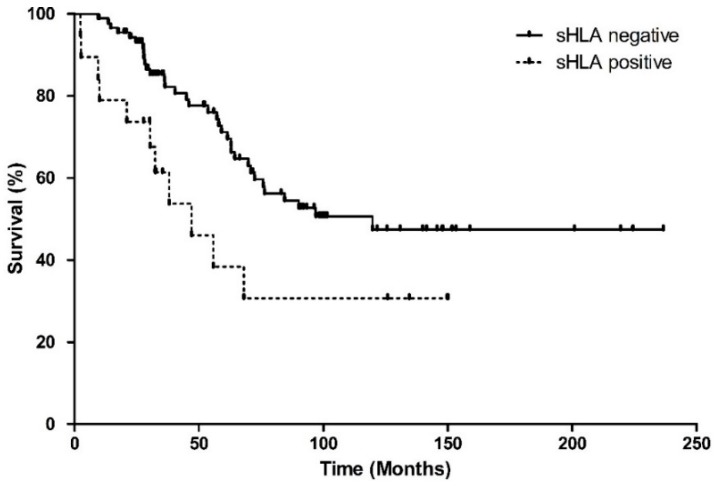
Kaplan–Meier survival curve showing melanoma-related survival, since enucleation, based on the sHLA expression in the aqueous humour of 108 UM patients. Curves represent the negative and positive sHLA groups. Both groups differ significantly in survival (Log Rank test *p* = 0.025).

Metastases occurred more frequently in the sHLA-positive group, with 13/19 (68%) patients developing metastases versus 36/88 (41%) patients in the negative group (*p* = 0.029, Table 1). The melanoma-related survival was significantly worse in the sHLA-positive group (Log-Rank, *p* = 0.025) (Figure 1).

### 2.3. Tumour HLA Expression and Infiltrate in the Tumour, Determined by a Fluidigm qPCR Assay

To determine HLA expression and infiltrate in UM tissue, a Fluidigm quantitative real-time PCR assay was performed on a subset of 89 tumours, of which the RNA was of sufficient quality. When comparing the tumours’ HLA-A, HLA-B and β_2_M expression between the sHLA-negative and sHLA-positive groups, no significant differences were found (*p* = 0.07, *p* = 0.46 and *p* = 0.60, respectively) (Table 2).

When comparing the sHLA-negative group versus the sHLA-positive group regarding the presence of infiltrate markers (CD40, CD8a, CD4, CD3e, CD163 and CD68) (Mann–Whitney U test), no significant difference between the two groups was observed (*p* = 0.38, *p* = 0.73, *p* = 0.52, *p* = 0.78, *p* = 0.52 and *p* = 1.00, respectively) (Table 2). This implies that we found no direct association between the sHLA expression in the aqueous humour and tumour infiltrate, as one would have expected. 

### 2.4. Tumour Location in Relation to sHLA in Aqueous Humour

We found no correlation between the tumour’s HLA expression and the presence of sHLA in the aqueous humour, nor any evidence for a relation between the tumour’s infiltrate profile and the presence of sHLA in aqueous humour. This made us wonder what else might determine the presence of sHLA. We already noticed that several clinical and genetic prognostic parameters were strongly associated with the presence of sHLA. We performed a multivariate regression analysis with data on chromosome 3 status, tumour prominence and involvement of the ciliary body, to see which characteristic is predominant in determining the presence of sHLA in the aqueous humour (Table 3). When combining chromosome 3 status, tumour prominence and involvement of the ciliary body in the multivariate model, both the involvement of the ciliary body and tumour prominence remained significant (*p* = 0.02 and *p* = 0.003, respectively).

## 3. Discussion

This is the first study describing the presence of sHLA in fluid of the anterior chamber. Due to the prognostic importance of an inflammatory phenotype in UM, we focused on determining whether the soluble form of cell-bound HLA Class I exists in the aqueous humour, and whether it corresponds to HLA expression on the tumor itself, and which clinical and histopathological characteristics of the UM determine its presence. We found measurable levels of sHLA in 19/108 of the aqueous humour samples from UM eyes, but no correlation with the level of expression of HLA in the primary tumour. However, there were other clear correlations; e.g., between tumour size and location, and with genetics: The sHLA-positive eyes contained tumours with a significantly larger prominence, which were more often located in the ciliary body and had a higher AJCC stage. With regard to chromosome status, the sHLA-positive eyes were significantly more often M3 tumours, with a gain of 8q, and loss of BAP1 protein expression. The association between involvement of the ciliary body and M3 is well known [11]. HLA expressing tumors may release proteins, and the involvement of the ciliary body especially may lead to leakage of sHLA and other proteins into the aqueous humor. Previous studies that focused on vitreous fluid found correlations between several cytokines and tumor prominence, as well as to T-cell infiltrate and macrophage infiltration [31]. 

Most studies on the presence of sHLA, in serum samples from metastatic cutaneous malignant melanoma patients, focus on the sHLA form of HLA-G and not on HLA-A/B [32]. Studies on HIV infection, autoimmune disease, but also cutaneous melanoma and renal cell carcinoma, have shown that sHLA levels in serum correlate to disease progression [33,34,35,36]. This aligns with our finding that more aggressive tumours especially, have positive levels of sHLA in the aqueous humor. As it was already hard to find sHLA in aqueous samples, we did not analyse serum samples from patients with an intraocular UM. A future study might look at serum from patients with UM metastases.

Current treatment options for metastatic UM are limited, but new therapies based on immunologic principles are being developed. One study on glioblastoma describes the potential of sHLA as the basis to identify potential biomarkers for disease detection and treatment [24]. sHLA molecules carry their original tumour-derived bound peptides. It was found that the immunopeptidomes of the sHLA molecules of cancer patients are very different with disproportionally-sized fractions of peptides originating from the tumour cells. It is hypothesised that this could help to identify tumour antigens, presented by the circulating sHLA complexes in the blood. Since UM spreads via the hematogenous route, plasma could be used to detect cancer biomarkers for early detection or monitoring [24]. One study on the topic of mass spectrometric identification investigated the capture of sHLA complexes and recovery of peptides from cell lines and serum of metastatic cutaneous melanoma patients. They found 972 different peptides in the sera of patients of which some were related to the melanocytic tumour, as previously described [37]. They stress that knowledge of the peptides may have important implications; e.g., in tumor rejection in immunotherapy.

Other studies focus on the mechanism by which sHLA plays a part in tumor-escape. It is thought that sHLA Class I molecules induce apoptosis of cytolytic effector cells; e.g., Natural Killer (NK) cells and CD8^+^ T lymphocytes [30,38,39].

A limitation of this study is the absence of a comparison of aqueous humor of UM eyes to normal eyes and eyes with other conditions. We tested a limited number of ocular fluids from normal eyes that underwent cataract surgery, and obtained negative results, which was expected as previous studies have shown low or undetectable levels of a wide range of cytokines in the normal aqueous humor [40]. Now that we have developed a test for sHLA, it will be interesting to expand testing of aqueous and vitreous for sHLA to eyes with different types of uveitis or with ocular lymphoma, as in both diseases, many different cytokines are being expressed in the aqueous humor [41].

Another limitation of this study is that the aqueous samples were only acquired from enucleated eyes, of which the tumours are usually large. It would be interesting to expand this analysis to eyes undergoing irradiation treatment. Additionally, as this assay detects the backbone of the HLA Class I molecule, while another addition would be to expand the assay to detect different HLA alleles to see whether they are expressed at the same level.

## 4. Materials and Methods

### 4.1. Patient Population

This study was conducted at the department of Ophthalmology, Leiden University Medical Center (LUMC), Leiden, The Netherlands. Aqueous humour samples were obtained from eyes of 108 UM patients who had been enucleated at the LUMC, between 1999 and 2017. Clinical patient information and data on survival were obtained from patient charts and the Dutch National Cancer Registry, and collected in a database. Follow-up time was defined as the time period between the date of enucleation and the moment of death or the last recorded follow-up moment. Data was updated in June 2019. Of the 108 patients, 45 had died from metastases, 19 from other causes and 44 were still alive at the end of follow-up.

Enucleated eyes underwent conventional histopathological evaluation by a pathologist specialised in ocular pathology. Tumour, lymph node and metastases (TNM) were staged in accordance with the 8th edition of the American Joint Committee on Cancer (AJCC) staging manual [42].

### 4.2. Collection of Aqueous Humour

Directly following enucleation, an anterior chamber puncture was performed to collect a sample of aqueous humour, using a 23 Gauge needle, and samples were directly stored at −80 °C. Additionally, a tumour sample was collected for cytogenetic analysis, as well as for DNA and/or mRNA analysis. Tumour tissue was fixed in 4% neutral buffered formalin for at least 48 h, dehydrated and embedded in paraffin.

### 4.3. Luminex Assay

Soluble HLA Class I in the anterior chamber fluid was measured using an in-house developed assay and optimised for use with aqueous humour. This assay uses magnetic beads coupled to anti-HLA-Class I directed, and purified W6/32 antibody (Department of Immunohematology, LUMC, The Netherlands), and the detection antibody anti- β_2_M-PE (BD Pharmingen; 551337). The Bio-Plex Luminex^TM^ system (Bio-Rad, Hercules, CA, USA) was used for read-out.

Measuring the levels of sHLA in aqueous humour by magnetic Luminex assay was newly developed, and is based on a previously-described assay [43]. First, aqueous humour samples were thawed and centrifuged for 4 min at 1400 rpm, after which samples were 1:1 diluted with 1% Bovine Serum Albumin/Phosphate Buffered-Saline (BSA/PBS) to obtain a concentration of 0.5% BSA/PBS. A calibration line with Dimer XI was prepared in 0.5% BSA/PBS. The beads were diluted to a concentration of 2500 beads per well in Assay Buffer (Diluent and Reagent kit III, Bio-Rad, Hercules, CA, USA) and washed twice with 80 µL wash water. Twenty microlitres of a calibration sample, a diluted test sample, or 0.5% BSA/PBS (blank) was added to each well of a 96-well plate, followed by incubation on a shaker, protected from light, at room temperature, for 45 min. Following three washes, the detection Ab (diluted 1:100 in detection Ab diluent) and 15 µL of this mixture were added to each well. The plate was incubated on a shaker, protected from light, at room temperature, for 30 min. Finally, the plate was washed three times with 80 µL wash buffer followed by adding 80 µL of assay buffer to each well. The plate was placed on a shaker until measurement (or stored at 4 °C). 

As this was the first time that aqueous humour samples were tested, optimal dilutions were determined first. The test values were interpolated in the calibration line. Samples below the lower limit of quantification were categorised as negative.

### 4.4. Chromosome Status and BAP1 Immunohistochemistry

For the 108 samples included in this study, three different cytogenetic tests were performed to determine the tumours’ chromosome statuses. On 36/108 tumours, both karyotyping and fluorescence in situ hybridisation (FISH) analysis with the use of DNA probes designed specifically for the centromere of chromosome 3, were performed. Twenty-three Tumours were analysed using a genome wide micro-array analysis of single-nucleotide polymorphisms (SNPs), performed with the Affymetrix 250K Nsp array (Affymetrix, Santa Clara, CA, USA), as previously described [44]. A total of 45 tumours was tested with all three techniques. In case of a disagreement between the three cytogenetic tests, tumours were defined as having monosomy 3 or a gain of 8q when any of the three before-mentioned tests showed this chromosomal aberration. Immunohistochemistry (IHC) of BAP1 was performed as previously described [44,45] and scored by an ocular pathologist. 

### 4.5. qPCR Analysis Using Fluidigm Array

A qPCR Fluidigm 96.96 dynamic array (FLUIDIGM Corporation, South San Francisco, CA, USA) was performed on tissue from 94 tumours. RNA isolation and quality checks were performed as previously described [46]. Total RNA (50–200 ng) was used for cDNA synthesis, following the manufacturer’s manuals. Ten-times diluted cDNA (1.25 µL) was pre-amplified containing 2.5 µL of Taqman Preamp master mix (Applied Biosystems, Foster City, CA, USA) and 1.25 µL of pooled primer mix for 14 cycles. The qPCR reactions were performed using Eva-green dye following the Fluidigm protocol, and results were collected on the BioMark HD system (FLUIDIGM). Absolute Cq values from duplicate measurements of each transcript were averaged, and relative gene expression levels were normalised to the geometric mean of the reference genes glyceraldehyde 3-phosphate dehydrogenase (GAPDH), β-actin, Hypoxanthine Phosphoribosyltransferase 1 (HPRT-1), Ribosomal Protein L13a (RPL13a) and Hydroxymethylbilane Synthase (HMBS).

### 4.6. Statistical Analyses

Statistical analyses were performed using SPSS software (IBM Corp. Released 2015. IBM SPSS Statistics for Windows, Version 23.0. Armonk, NY, USA). The Pearson chi-square test was used to analyse categorical data and a Linear-by-Linear Association was used in case of more than two categories. The Mann–Whitney U Test was used as a non-parametric test to compare two groups. For multivariate analysis, a multivariate linear regression was conducted. Kaplan–Meier curves were made to analyse survival, with the log rank test to compare groups and were drawn using GraphPad Prism version 5.00 for Windows, GraphPad Software, La Jolla, CA, USA. *p* values of <0.05 were considered statistically significant. 

### 4.7. Study Approval

This study was approved by the Biobank Committee of the LUMC (number: Uveamelanoomlab-2019-5, date of approval: 20th May 2019). The research adhered to Dutch law and the tenets of the Declaration of Helsinki (World Medical Association of Declaration 2013; ethical principles for medical research involving human subjects).

## 5. Conclusions

Soluble HLA is especially present in the aqueous humour of large tumours, which display ciliary body involvement, loss of chromosome 3, and negative BAP1 staining, and which have a higher chance of developing metastases. As sHLA is related to metastasis development, the presence of detectable levels of sHLA in the aqueous humour may aid in selecting cases for future adjuvant trials or aid in prognostication, without having to take tumour material from mateiral or a tumour biopsy.

## Figures and Tables

**Table 1:** Characteristics of patients with a uveal melanoma (UM) that underwent enucleation in the Leiden University Medical Center (LUMC) between 1999 and 2016 (*n* = 108), categorised as soluble HLA (sHLA) negative or sHLA positive, based on the level of sHLA in the aqueous humour.

Clinical and Histopathologic Characteristics	Patients, *n* (%) ^a^		
	sHLA Negative (*n* = 89)	sHLA Positive (*n* = 19)	*p*-Value *
**Gender**			0.09 ^b^
Female	33 (37)	11 (58)	
Male	56 (63)	8 (42)	
**Previous tumour treatment prior to enucleation**			0.69 ^d^
No	86 (97)	18 (95)	
Yes	3 (3)	1 (5)	
**Eye**			0.28 ^b^
OD	52 (58)	7 (37)	
OS	37 (42)	12 (63)	
**Age at enucleation**			0.46 ^e^
in years, mean (±S.D.)	59 (±14)	63 (±16)	
**Eye colour ^c^ (known in 58 cases)**			0.056 ^b^
Light	49 (88)	10 (67)	
Dark	7 (12)	5 (33)	
**Largest basal tumour diameter**			0.40 ^e^
in mm, mean (±S.D.) (known in 107 cases)	12.3 (±3.1)	13.1 (±3.1)	
**Tumour prominence**			<0.001 ^e,^*
in mm, mean (±S.D.) (known in 107 cases)	7.0 (±2.9)	9.8 (±2.2)	
**Ciliary body involvement**			<0.001 ^b,^*
No	64 (72)	5 (26)	
Yes	25 (28)	14 (74)	
**Mitotic count ^f^ (known in 101 cases)**			0.44 ^e^
mean (±S.D.)	4.7 (±3.3)	6.4 (±7.1)	
**Histopathologic cell type**			0.064 ^b^
Spindle cell	28 (32)	2 (11)	
Epithelioid or mixed cell type	61 (69)	17 (89)	
**Bruch’s membrane (known in 104 cases)**			0.076 ^d^
Unclear	10 (12)	5 (26)	
Intact	21 (25)	1 (5)	
Broken	53 (62)	13 (68)	
**AJCC Stage, 8th edition**			<0.001 ^d,^*
I	11 (14)	0 (0)	
IIA	29 (36)	0 (0)	
IIB	23 (29)	6 (40)	
IIIA, IIIB, IIIC	17 (21)	8 (53)	
IV	0 (0)	1 (7)	
**Monosomy 3 status (known in 103 cases)**			0.034 ^b,^*
D3	37 (44)	3 (17)	
M3	48 (57)	15 (83)	
**8q status (known in 95 cases)**			0.003 ^b,^*
No gain of 8q	35 (45)	1 (6)	
Gain of 8q	43 (55)	16 (94)	
**Duplication of chromosome 6p (known in 82 cases)**			0.98 ^b^
No gain of 6p	31 (46)	7 (37)	
Gain of 6p	36 (54)	8 (42)	
**Nuclear BAP1 staining (known in 97 cases)**			0.036 ^b,^*
Normal	29 (37)	2 (11)	
Absent	50 (63)	16 (89)	
**Metastases (known in 107 cases)**			0.029 ^b,^*
Absent	52 (59)	6 (32)	
Present	36 (41)	13 (68)	
**Vital status**			0.36 ^d^
Dead due to UM metastases	34 (38)	11 (58)	
Average follow-up time, months	50	27	
Dead due to other cause	19 (21)	0 (0)	
Alive at last follow-up date	36 (40)	8 (42)	

* Test is significant at the 0.05 level (2-tailed). Significant *p* values are shown in bold. ^a^ Percentages are rounded and may not total 100; ^b^ Pearson χ^2^ test; ^c^ light eye colours: blue, grey, green, hazel—dark eye colour: brown; ^d^ linear-by-linear association; ^e^ Mann–Whitney U Test; ^f^ number of mitoses per mm^2^ with 40× magnification, in eight high power fields.

**Table 2:** Expression of different HLA- and infiltrate-related markers. Presence or absence of soluble HLA (sHLA) in the aqueous humor of UM-containing eyes compared to the expression of HLA and infiltrate related markers as determined by qPCR on primary uveal melanoma-tissue. The median is displayed, with the 95% bootstrap Confidence Intervals (CI).

sHLA			
Fluidigm Marker	Negative (Median, 95% CI)	Positive (Median, 95% CI)	*p*-Value *^,§^
HLA-A, *n* = 80	431 (218–834)	1264 (172–2162)	0.07
HLA-B, *n* = 81	123,245 (81,117–266,604)	205,282 (55,537–1,062,673)	0.46
β_2_M, *n* = 70	373,244 (226,274–566,287)	416,625 (22,3013–1,391,012)	0.60
CD40, *n* = 82	670 (451–1454)	516 (372–869)	0.38
CD8a, *n* = 69	34 (18–147)	182 (15–1027)	0.73
CD4, *n* = 71	274 (163–347)	188 (95–380)	0.52
CD3e, *n* = 65	116 (79–213)	221 (43–1212)	0.78
CD163, *n* = 79	1023 (509–2000)	839 (484–2404)	0.52
CD68, *n* = 81	4934 (2989–9072)	5473 (2000–25,873)	1.00

^§^ Mann–Whitney U test; * test is significant at the 0.05 level (2-tailed).

**Table 3:** Univariate and multivariate linear regression analysis on important prognostic tumour characteristics and sHLA expression in the aqueous humour of UM eyes (n = 108). B stands for the unstandardised regression coefficient and Beta (β) is the standardised regression coefficient. The β indicates the effect that a change to the standard deviation (SD) of the independent variable has on the dependent variable.

sHLA			
Variables	B (95% CI)	Beta (β)	*p*-Value
**Univariate regression**			
Chromosome 3 status (*n* = 103)	0.17 (0.01–0.31)	0.21	0.034 *
Involvement of ciliary body (*n* = 108)	2.87 (−0.43–−0.14)	−0.36	<0.001 *
Tumour prominence (*n* = 107)	0.045 (0.02–0.07)	0.36	<0.001 *
**Multivariate regression**			
Model 1			
Chromosome 3 status	0.097 (−0.53–0.25)	0.13	0.20
Involvement of ciliary body	−0.24 (−0.39–−0.86)	−0.30	0.002 *
Model 2			
Tumour prominence	0.035 (0.01–0.06)	0.27	0.003 *
Involvement of ciliary body	−0.233 (−0.38–−0.09)	−0.29	0.002 *
Model 3			
Chromosome 3 status	0.44 (0.02–0.07)	0.197	0.035 *
Tumour prominence	0.15 (0.01–0.30)	0.348	<0.001 *
Model 4			
Chromosome 3 status	0.10 (−0.46–0.25)	0.13	0.17
Tumour prominence	0.04 (0.01–0.06)	0.28	0.003 *
Involvement of ciliary body	−0.18 (−0.34–−0.03)	−0.23	0.021 *

* Significant *p*-values are shown in bold.
